# Implantação de Marca-passo em Pacientes com Doença de Chagas: Estudo Caso-Controle de Fatores Contextuais Associados e Prognóstico

**DOI:** 10.36660/abc.20250003

**Published:** 2026-03-04

**Authors:** Sâmara Fernandes Leite, Maria Carmo Pereira Nunes, Cesar Augusto Taconeli, Mathaus Henrique Reis Silva, Dardiane Santos Cruz, Lea Campos Oliveira, Ester Cerdeira Sabino, Marco Paulo Tomáz Barbosa, Antonio Luis Pinho Ribeiro, Ariela Mota Ferreira, Desirée Sant’Ana Haikal

**Affiliations:** 1 Programa de Pós Graduação em Ciências da Saúde Universidade Estadual de Montes Claros MG Brasil Programa de Pós Graduação em Ciências da Saúde, Universidade Estadual de Montes Claros (UNIMONTES), MG – Brasil; 2 Departamento de Clínica Médica Faculdade de Medicina Universidade Federal de Minas Gerais Belo Horizonte MG Brasil Departamento de Clínica Médica, Faculdade de Medicina, e Centro de Telessaúde e Serviço de Cardiologia e Cirurgia Cardiovascular, Hospital das Clínicas, Universidade Federal de Minas Gerais, Belo Horizonte, MG – Brasil; 3 Departamento de Estatística Universidade Federal do Paraná Curitiba PR Brasil Departamento de Estatística, Universidade Federal do Paraná, Curitiba, PR – Brasil; 4 Instituto de Medicina Tropical Faculdade de Medicina Universidade de São Paulo São Paulo SP Brasil Instituto de Medicina Tropical da Faculdade de Medicina da Universidade de São Paulo – USP,São Paulo, SP – Brasil; 5 Serviço de Cardiologia e Cirurgia Cardiovascular Hospital das Clínicas Universidade Federal de Minas Gerais Belo Horizonte MG Brasil Serviço de Cardiologia e Cirurgia Cardiovascular, Hospital das Clínicas, Universidade Federal de Minas Gerais, Belo Horizonte, MG – Brasil

**Keywords:** Doença de Chagas, Marca-Passo Artificial, Cardiomiopatia Chagásica, Mortalidade, Sobrevida

## Abstract

**Fundamento:**

A cardiomiopatia chagásica crônica (CCC) é a manifestação mais grave da doença de Chagas e pode apresentar bradiarritmias que requerem implante de marca-passo (MP).

**Objetivo:**

1. Identificar fatores contextuais associados à implantação de MP em indivíduos com CCC. 2. Avaliar o prognóstico (óbito) nesses grupos durante um período de acompanhamento de 4 anos.

**Métodos:**

1. Estudo caso-controle aninhado na coorte SaMi-Trop. Os casos (pacientes com CCC que receberam implante de MP entre as ondas 1 e 2 do estudo) foram pareados com controles na proporção de 1:3, considerando equivalência quanto à gravidade cardíaca basal. Foram consideradas variáveis independentes relacionadas ao contexto municipal. Foi realizada regressão logística binária. 2. Análise de sobrevida para avaliar o prognóstico desses grupos, ajustada para variáveis clínicas (regressão de Cox), considerando o óbito como desfecho. As conclusões foram baseadas em um nível de significância de 5%.

**Resultados:**

O estudo incluiu 46 casos e 138 controles. As chances de implantação de MP foram maiores entre indivíduos residentes em municípios com: maior população (razão de chances [OR]: 3,6; intervalo de confiança [IC] de 95%: 1,3 a 9,6), maior cobertura da Estratégia Saúde da Família (OR: 5,3; IC 95%: 1,8 a 15,8) e menor número de eletrocardiógrafos por mil habitantes (OR: 2,6; IC 95%: 1,0 a 6,6). Considerando 4 anos de seguimento, o óbito foi observado em 32,6% dos casos e 21,2% dos controles. Apenas a fração de ejeção afetou a sobrevida (risco relativo: 7,2; IC 95% 3,6 a 14,2).

**Conclusão:**

Fatores contextuais interferiram na implantação de MP. No entanto, a implantação de MP não influenciou o óbito durante os 4 anos de seguimento, o qual esteve associado apenas à fração de ejeção.

## Graphical abstract

**Figura Central:**
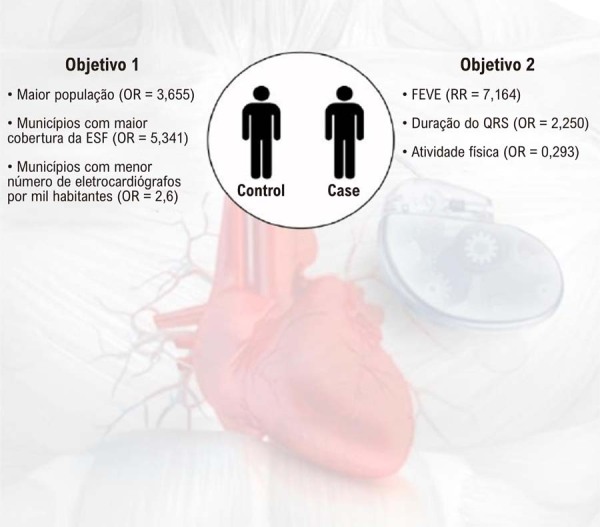
Implantação de Marca-passo em Pacientes com Doença de Chagas: Estudo Caso-Controle de Fatores Contextuais Associados e Prognóstico

ESF: Estratégia Saúde da Família; FEVE: fração de ejeção do ventrículo esquerdo; OR: razão de chances; RR: risco relativo.

## Introdução

A doença de Chagas (DC) é uma infecção parasitária sistêmica causada pelo *Trypanosoma cruzi* ( *T. cruzi* ), que afeta predominantemente populações pobres e vulneráveis.^1^ É endêmica em 21 países da América Latina, onde aproximadamente 6 milhões de pessoas são afetadas pela doença.^2^ Apresenta uma incidência anual de 30.000 novos casos e uma mortalidade anual de 14.000 óbitos na América Latina.^3 , 4^ No Brasil, as estimativas variam de 1,0% a 2,4% da população infectada com *T. cruzi* .^5^

A cardiomiopatia chagásica crônica (CCC) é considerada a manifestação mais importante e grave da DC crônica.^6^ É responsável pela alta morbimortalidade da doença, incapacidade permanente, bem como significativo impacto econômico e social.^1 , 7^

A CCC afeta aproximadamente 20% a 30% dos indivíduos infectados por *T. cruzi* .^8 - 10^ Pode apresentar alterações eletrocardiográficas significativas, como bloqueio atrioventricular de segundo e terceiro graus, bradicardia e fibrilação atrial,^11 , 12^ bem como alterações bioquímicas, como o biomarcador de insuficiência cardíaca peptídeo natriurético tipo B N-terminal (NT-proBNP), associado ao diagnóstico de CCC e diferentes cardiomiopatias.^12 - 14^ Nessas situações, especialmente para o tratamento de bradiarritmias sintomáticas e em associação a outras avaliações clínicas, a implantação de marca-passo (MP) pode ser a terapia de escolha.^6 , 7^

Acredita-se que características contextuais do ambiente em que vivem indivíduos com DC possam influenciar o acesso à implantação de MP. Além disso, há escassez na literatura de estudos longitudinais que acompanhem a progressão de pacientes gravemente acometidos por CCC, comparando aqueles que tiveram e os que não tiveram acesso à implantação de MP.

Portanto, o presente estudo visou: 1. Identificar fatores contextuais associados à implantação de MP entre pacientes com CCC, pareados pela gravidade do comprometimento cardíaco ao longo de 2 anos de seguimento (estudo caso-controle); 2. Avaliar a relação entre a implantação de MP e a sobrevida nesses mesmos grupos, com e sem implantação de MP, ao longo de 4 anos de seguimento (estudo prospectivo).

## Método

### Desenho do estudo

Para o presente estudo, foram utilizados dados da coorte SaMi-Trop. Foi realizado um estudo caso-controle, seguido de uma análise prospectiva de sobrevida.

O SaMi-Trop é um estudo de coorte multicêntrico envolvendo indivíduos com DC, conduzido por meio de uma parceria entre quatro universidades públicas brasileiras. É financiado pelos National Institutes of Health (NIH) dos Estados Unidos (números de bolsa: P50510 AI098461-02 e U19AI098461-06). O estudo acompanha aproximadamente 2.000 indivíduos com DC de 21 municípios em duas regiões endêmicas para a doença no estado de Minas Gerais desde 2013.^15 , 16^

O estudo SaMi-Trop compreendeu três ondas de coleta de dados. A primeira onda (linha de base), que ocorreu entre 2013 e 2014, incluiu 2.157 indivíduos com DC com 18 anos ou mais. A segunda onda ocorreu entre 2015 e 2016, com 1.709 participantes restantes. Finalmente, a terceira onda ocorreu entre 2019 e 2021, com 1.115 participantes restantes. Em todas as avaliações, foram realizadas entrevistas, coleta de sangue e eletrocardiograma (ECG) de 12 derivações, sendo acrescentado o exame ecocardiográfico nos acompanhamentos. A entrevista abordou dados sociodemográficos, hábitos de vida, atividade física, aspectos de qualidade de vida, histórico médico e tratamento da DC.

### Aspectos éticos

A aprovação ética foi obtida do comitê de ética competente (CEP/USP 042/2012, UNIMONTES 2.474.172 e CONEP 179.685). Todos os indivíduos concordaram em participar do presente estudo e assinaram o termo de consentimento livre e esclarecido antes do início do estudo.

### Estudo caso-controle (primeiro objetivo)

#### Seleção de participantes

Utilizando dados dos participantes da coorte SaMi-Trop, foram formados dois grupos de análise (caso versus controle), considerando como potenciais participantes apenas os indivíduos com sorologia confirmada para DC por meio de ensaio imunoenzimático (ELISA) que apresentaram envolvimento cardíaco na primeira onda de avaliação (critérios apresentados abaixo).

Indivíduos “casos” foram definidos como aqueles que realizaram implantação de MP entre a primeira e a segunda ondas de avaliação. Indivíduos “controles” foram definidos como aqueles que não realizaram implantação de MP entre a primeira e a segunda onda, mas apresentaram acometimento cardíaco durante a primeira onda, de forma semelhante aos casos (pareamento).

Foi realizado um pareamento manual de 1:3 (caso:controle) considerando as variáveis de acometimento cardíaco, sexo (masculino versus feminino) e idade categorizada pela média (até 60 anos versus 61 anos ou mais). O acometimento cardíaco foi definido como: bloqueio atrioventricular de segundo grau e de terceiro grau, fibrilação atrial, bradicardia sinusal, todos categorizados como não versus sim; classe funcional categorizada como sem alteração (classe 1) versus com alteração (classes 2, 3 e 4), de acordo com a New York Heart Association (NYHA),^17^ e nível de NT-proBNP categorizado por idade (não alterado versus alterado).^13^

#### Variável dependente

A variável dependente foi relacionada à implantação de MP entre a primeira e a segunda onda de seguimento (caso versus controle), obtida por meio de perguntas da entrevista e confirmada por ECG.

#### Contextuais e municipais

A coleta de dados contextuais foi realizada para caracterizar os aspectos sociais, demográficos e de serviços de saúde dos 21 municípios participantes da coorte SaMi-Trop. Oito variáveis contextuais foram consideradas, coletadas em plataformas e sistemas de informação de saúde pública. As informações foram coletadas com referência ao ano da primeira onda de avaliação da coorte (2013 a 2014), buscando os dados disponíveis mais próximos a essa referência. O Material Suplementar 1 apresenta informações sobre as variáveis contextuais consideradas.

A variáveis de Índice de Desenvolvimento Humano Municipal (IDHM) e Índice de Desempenho do Sistema Único de Saúde (IDSUS) foram coletadas e categorizadas de acordo com padrões internacionais e nacionais e, posteriormente, dicotomizadas. Os pontos de corte adotados foram “baixo” (0 a 0,55) versus “médio/alto” (0,556 a 0,699/0,700 a 0,799) e “0,50 a 0,59” versus “0,60 a 0,79” para o IDH e o IDSUS, respectivamente. As variáveis de população, índice de Gini, gasto total com saúde per capita, cobertura da Estratégia Saúde da Família (ESF), distância do centro de referência e número de eletrocardiógrafos por mil habitantes foram coletadas numericamente e subsequentemente dicotomizadas, adotando-se o 25º percentil ou o 75º percentil como ponto de corte, dependendo se a variável representava uma medida negativa (valores baixos indicando situação melhor) ou positiva (valores altos indicando situação melhor). O objetivo era separar 25% dos municípios em situações melhores de 75% dos municípios em situações piores, dado que os municípios incluídos apresentavam perfis geralmente semelhantes e predominantemente precários ( Tabela 1 ). A variável de presença de cardiologista foi coletada e categorizada como presente versus ausente.

**Tabela 1:** – Distribuição das variáveis de pareamento entre os grupos caso (com marca-passo) e controle (sem marca-passo). Pacientes com doença de Chagas. Minas Gerais, Brasil (2013 a 2016)

Características	Descritivas	Bivariadas		Valor p**
	n (%)	Controle n=138	Caso n=46	
**Variáveis de pareamento** Bloqueio AV de segundo grau*				
Negativo	180 (98,4%)	136 (98,6%)	44 (97,8%)	0,723
Positivo	3 (1,6%)	2 (1,4%)	1 (2,2%)	
Bloqueio AV de terceiro grau*				
Negativo	182 (99,5%)	138 (100%)	44 (97,8%)	0,079
Positivo	1 (0,5%)	0 (0,0%)	1 (2,2%)	
FA*				
Negativo	176 (96,2%)	132 (95,7%)	44 (97,8%)	0,519
Positivo	7 (3,8%)	6 (4,3%)	1 (2,2%)	
Bradicardia sinusal*				
Negativo	176 (96,2%)	133 (96,4%)	43 (95,6%)	0,803
Positivo	7 (3,8%)	5 (3,6%)	2 (4,4%)	
**Classe funcional**				
Sem alteração da classe funcional	78 (42,4%)	58 (42%)	20 (43,5%)	0,863
Classe funcional com alteração (2, 3 e 4)	106 (57,6%)	80 (58%)	26 (56,5%)	
Nível de NT-proBNP*				
Sem alteração	140 (76,5%)	104 (75,9%)	36 (78,3%)	0,745
Com alteração	43 (23,5%)	33 (24,1%)	10 (21,7%)	
**Sexo**				
Masculino	64 (34,8%)	47 (34,1%)	17 (37%)	0,721
Feminino	120 (65,2%)	91 (65,9%)	29 (63%)	
**Idade**				
Até 60 anos	87 (47,3%)	67 (48,6%)	20 (43,5%)	0,551
61 anos ou mais	97 (52,7%)	71 (51,4%)	26 (56,5%)	

AV: atrioventricular; FA: fibrilação atrial. * Variação total de n de 184. ** Teste qui-quadrado de Pearson.

## Análise estatística

As análises descritivas e bivariadas (teste qui-quadrado de Pearson) das variáveis de pareamento foram conduzidas para confirmar a similaridade entre os grupos de casos e controles. O efeito da implantação de MP no tempo de sobrevida dos participantes foi analisado utilizando técnicas de análise de sobrevida. O método de Kaplan-Meier foi utilizado para ajustar as curvas de sobrevida e realizar uma comparação preliminar entre os grupos, e o teste de log-rank para comparar os tempos de sobrevida.

Posteriormente, iniciou-se a modelagem múltipla utilizando regressão logística binária tradicional. Todas as variáveis contextuais foram inicialmente incluídas na análise, e foram mantidas como fatores de ajuste as variáveis renda, alfabetização e cor da pele autodeclarada. As variáveis contextuais foram ajustadas manualmente utilizando o método *stepwise backward* , considerando um nível de significância de 5%. O teste de Hosmer-Lemeshow foi empregado para avaliar a qualidade do ajuste do modelo logístico. A porcentagem da variância explicada pelo modelo foi estimada utilizando o Pseudo R^2^ de Nagelkerke. As análises foram conduzidas utilizando o software Predictive Analytics SoftWare (PASW/SPSS)^®^, versão 24.0 para Windows^®^.

## Estudo prospectivo (segundo objetivo)

### Participantes do estudo

Os mesmos grupos de análise selecionados na segunda onda de avaliação foram considerados: casos (com implantação de MP) versus controles (sem implantação de MP). Indivíduos perdidos no seguimento e indivíduos do grupo controle que foram submetidos à implantação de MP após a segunda onda de avaliação foram excluídos das análises.

### Variável dependente

O evento de interesse foi o óbito (não versus sim). A variável dependente considerada foi o tempo decorrido desde a segunda onda de coleta de dados até a ocorrência do óbito ou até o final do período de seguimento (terceira onda), aproximadamente 2.200 dias (6 anos).

O óbito foi considerado para os indivíduos para os quais essa informação estava disponível (por meio de familiares ou serviços de saúde), ou quando foi identificado registro no Sistema Nacional de Mortalidade, onde foram realizadas buscas para todos os participantes perdidos.

### Variáveis independentes

Para as variáveis independentes, considerou-se a categorização caso versus controle estabelecida no estudo, juntamente com um conjunto de variáveis clínicas, sociodemográficas e comportamentais, incluindo a fração de ejeção do ventrículo esquerdo (FEVE), obtida por ecocardiograma e categorizada como: normal (≥ 50%) versus alterada (< 50%) %).^18^ A variável duração do complexo QRS foi categorizada como: normal (n < 120 ms) versus alterada (≥ 120 ms).^18^ Diabetes mellitus e hipertensão permaneceram como coletadas: não versus sim. A autopercepção de saúde foi obtida por meio do autorrelato do participante durante a entrevista por meio da pergunta “Como você avaliaria sua saúde hoje?”. Foi adotada uma escala Likert com opções de resposta, posteriormente dicotomizada como: positiva (boa, muito boa e regular) versus negativa (ruim e muito ruim). A renda per capita foi categorizada em relação à metade do salário mínimo vigente na época da coleta de dados (2014): acima de R$ 362,01 versus até R$ 362,00. A alfabetização foi avaliada por meio da pergunta: “Você sabe ler e escrever?”, categorizada como: não versus sim. O estado civil foi categorizado como: união estável (coabitando com parceiro/a, casado/a) versus sem união estável (solteiro/a, viúvo/a e outros). A atividade física foi avaliada por meio da pergunta: “Você pratica alguma atividade física?” e categorizada como: não versus sim.

## Análise estatística

O efeito da implantação de MP no tempo de sobrevida dos participantes foi analisado utilizando técnicas de análise de sobrevida. O método de Kaplan-Meier foi empregado para ajustar as curvas de sobrevida e realizar uma comparação preliminar entre os grupos. Posteriormente, foi realizada uma análise de regressão para estimar e testar o efeito de interesse. Nessa etapa, foi utilizado o modelo de riscos proporcionais de Cox, considerando, além do status de implantação de MP, o seguinte conjunto de variáveis: fração de ejeção, duração do QRS, diabetes, hipertensão, autopercepção de saúde, renda, alfabetização, estado civil e atividade física. Adicionalmente, o efeito do desenho experimental (caso-controle) foi incorporado ao modelo por meio de um efeito aleatório (termo de fragilidade) com distribuição gama, a fim de acomodar a correlação intragrupo e a variabilidade entre grupos induzidas pelo desenho. Inicialmente, foi realizada uma análise bivariada para avaliar o efeito da implantação de MP e de outras variáveis não ajustadas pelas demais sobre o tempo de sobrevida. Variáveis significativas ao nível de 20% foram consideradas para inclusão no modelo de regressão múltipla. Após o ajuste do modelo de regressão múltipla, as variáveis não significativas ao nível de 5% foram sucessivamente removidas do modelo. Os resultados são apresentados na forma de riscos relativos (RR) e intervalos de confiança (IC) de 95%, juntamente com seus respectivos níveis de significância. A interação entre o tratamento (MP) e a fração de ejeção também foi investigada para avaliar possíveis diferenças no efeito do tratamento em participantes com diferentes níveis de fração de ejeção. As conclusões foram baseadas em um nível de significância de 5%. Todas as análises foram realizadas utilizando o software estatístico R, versão 4.2.1. A biblioteca survival foi utilizada para a análise de sobrevida.^19^

## Resultados

**Figura 1:**
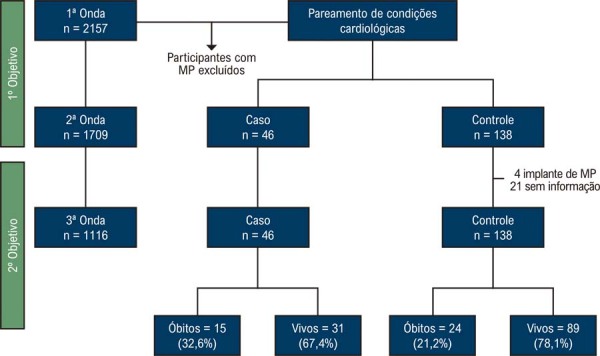
– Fluxograma dos participantes classificados como caso e controle, perdas de seguimento e ocorrência de óbito.

Entre os 1.709 indivíduos com DC avaliados na segunda onda da coorte SaMi-Trop, foram identificados 46 casos e 138 controles, totalizando 184 indivíduos ( Figura 1 ).

Considerando as variáveis de pareamento, não houve diferença estatística entre casos e controles ( Tabela 1 ), indicando similaridade entre os grupos.

### Primeiro objetivo

Entre as variáveis contextuais consideradas para avaliar a associação com a implantação de MP, observou-se que a maioria dos indivíduos residia em municípios com menor população (67,4%), maior IDHM (78,8%), maior coeficiente de Gini (91,3%), melhor IDSUS (66,3%), menor gasto total com saúde por habitante (89,7%), maior cobertura da EFS (57,6%), sem cardiologista no serviço de saúde (81%), mais distantes do centro de referência em saúde especializado (87,5%) e com menor número de eletrocardiógrafos por mil habitantes (65,8%).

O modelo de regressão múltipla ajustado revelou uma maior probabilidade de implantação de MP entre aqueles que residiam em municípios com maior população (OR: 3,655; IC 95%: 1,385 a 9,650), em municípios com maior cobertura da EFS (OR: 5,341; IC 95%: 1,805 a 15,804) e em municípios com menor número de eletrocardiógrafos por mil habitantes (OR: 2,6; IC 95%: 1,057 a 6,620) ( Tabela 2 ). Os fatores contextuais foram associados ao acesso à implantação de MP ( Figura Central ).

**Tabela 2:** – Modelo de regressão logística binária múltipla de fatores contextuais associados à implantação de marca-passos artificiais em indivíduos da coorte SaMi-Trop. Minas Gerais, Brasil. n = 184

			Fatores contextuais associados à implantação de marca-passo	
Variáveis	Controle	Caso	OR [IC 95%]	Valor p
**População**				
População menor	96 (69,6%)	28 (60,9%)	1	0,009
População maior	42 (30,4%)	18 (39,1%)	3,655 [1,385-9,650]	
**Cobertura da ESF**				
Cobertura inferior a 100%	62 (44,9%)	16 (34,8%)	1	0,002
Cobertura de 100%	76 (55,1%)	30 (65,2%)	5,341 [1,805-15,804]	
**Número de eletrocardiógrafos por mil habitantes**				
Acima de 0,21	49 (35,5%)	14 (30,4%)	1	0,038
Abaixo de 0,20	89 (64,5%)	32 (69,6%)	2,645 [1,057-6,620]	
Valor p do teste de Hosmer-Lemeshow = 0,513 Pseudo R^2^ (Nagelkerke): 10,3% Modelo ajustado para renda, alfabetização e cor da pele				

ESF: Estratégia Saúde da Família.

### Segundo objetivo

Em relação ao segundo objetivo, observou-se o óbito de 15 (32,6%) indivíduos entre os casos e 24 (21,2%) entre os controles ( Figura 1 ). Entre os indivíduos classificados como controles, ocorreram 25 óbitos, e, portanto, a participação de 113 controles foi considerada nesta análise (segundo objetivo).

**Figura 2:**
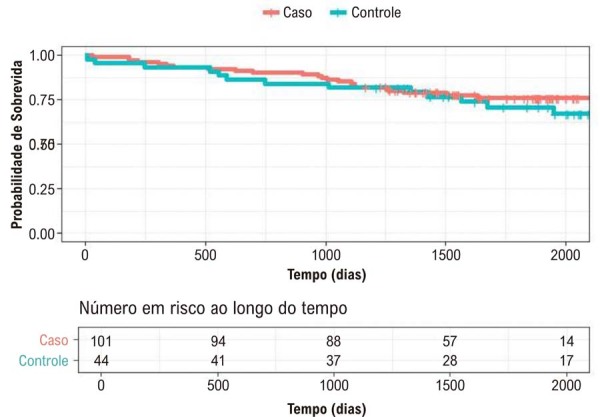
– Curva de Sobrevida de Kaplan-Meier dos Grupos Caso versus Controle.

A Figura 2 apresenta as curvas de sobrevida para os grupos de casos e controles (p > 0,05).

Na análise bivariada em relação ao óbito, variáveis como FEVE, duração do QRS e atividade física foram associadas ao desfecho. Na regressão de Cox, apenas a variável FEVE (RR: 7,164; IC 95%: 3,615 a 14,197) afetou significativamente a sobrevida de indivíduos com DC ( Figura Central ). O MP não esteve associado ao desfecho ( Tabela 3 ).

**Tabela 3:** – Análise bivariada e modelo de regressão múltipla de Cox da relação entre implantação de marca-passo e prognóstico (óbito) nos grupos, ao longo de 4 anos de seguimento. Regiões endêmicas do Estado de Minas Gerais, Brasil. n = 113

Covariáveis	Risco relativo [IC 95%]	Valor p	Modelo de regressão múltipla, risco relativo [IC 95%]	Valor p
**Categorização caso-controle**				
Caso	1,437 [0,717 - 2,877]	0,425	1,408 [0,710-2,793]	0,333
**Fração de ejeção**				
Com alteração	7,164 [3,615- 14,197]	≤ 0,001	7,201[3,632-14,277]	≤ 0,001
**Duração do QRS**				
Com alteração	2,350 [1,103 -5,007]	≤ 0,001	-	-
**Diabetes**				
Sim	0,853 [0,310-2,347]	0,997	-	-
**Hipertensão**				
Sim	1,126 [0,518-2,449]	0,927	-	-
**Percepção de saúde**				
Negativa	1,903 [0,926-3,908]	0,061	-	-
**Renda**				
Abaixo de R$ 362.00	0,975 [0,475-2,002]	0,738	-	-
**Alfabetização**				
Sim	0,709 [0,339-1,481]	0,222	-	-
**Estado civil**				
União estável	1,185 [0,569-2,467]	0,755	-	-
**Prática de atividade física**				
Sim	0,293 [0,086-0,996]	0,027	-	-

O Material Suplementar 2 apresenta as curvas de sobrevida considerando as seguintes variáveis: FEVE, duração do complexo QRS, percepção autorrelata da saúde e prática de atividade física.

O teste de log-rank foi utilizado para comparar os tempos de sobrevida entre os grupos. De acordo com os resultados do teste, não foi observada diferença entre os tempos de sobrevida dos pacientes nos grupos caso e controle (p = 0,403). No entanto, foram identificadas diferenças significativas para diferentes níveis de fração de ejeção (p < 0,001), percepção de saúde (p = 0,010), duração do QRS (p = 0,048) e atividade física (p = 0,045). As curvas de sobrevida estimadas pelo método de Kaplan-Meier, apresentadas no Material Suplementar 2, indicaram tempos de sobrevida mais longos para pacientes com FEVE acima de 50%, percepção de saúde positiva, duração normal do QRS e prática de atividade física.

A interação entre MP e FEVE também foi investigada para avaliar uma possível diferença no efeito do tratamento para pacientes com diferentes níveis de fração de ejeção. No entanto, não foi observado nenhum efeito significativo para essa interação (p = 0,39).

## Discussão

Os resultados do presente estudo demonstraram que, ao parear indivíduos com DC e acometimento cardíaco semelhante, fatores contextuais foram associados ao acesso à implantação de MP. Residir em municípios com populações maiores, municípios com maior cobertura da EFS e municípios com menos eletrocardiógrafos por mil habitantes aumentou a probabilidade de acesso à implantação de MP. Além disso, ao seguir esses mesmos indivíduos com DC e acometimento cardíaco semelhante, observou-se que ter ou não acesso à implantação de MP não influenciou a mortalidade ao longo de 4 anos. A mortalidade foi associada apenas à alteração da FEVE.

Apesar da reconhecida relevância dos fatores contextuais em doenças crônicas,^19 - 21^ existe uma lacuna na literatura em relação à implantação de MP em pacientes com DC crônica, especialmente considerando as características do contexto em que vivem. A influência de variáveis que refletem as condições de saúde na implantação de MP já foi estabelecida na literatura.^10^ No entanto, é necessário considerar que informações relacionadas ao contexto em que se vive influenciam as condições de saúde dos pacientes.^19 - 21^

Observou-se que residir em municípios com maior população aumentou a chance de acesso à implantação de MP entre os participantes. Inicialmente, esperava-se esse resultado, uma vez que os municípios maiores tendem a ter acesso mais fácil a cuidados de saúde especializados e a apresentar melhores indicadores de saúde e atendimento.^23 , 24^

Indivíduos residentes em municípios com maior cobertura da ESF apresentaram maior probabilidade de acesso à implantação de MP. A ESF desempenha um papel crucial no manejo de pacientes com DC, uma vez que está estrategicamente posicionada em relação a outros pontos de atenção à saúde.^25^ Por ser o serviço de saúde mais próximo dessa população e estar presente em áreas rurais e remotas, pode ser considerado um ator-chave no cuidado do paciente, facilitando o acesso à atenção e ao acompanhamento especializados desses pacientes, especialmente em áreas menos favorecidas.^26 , 27^ A ESF absorve grande parte das demandas de saúde de uma comunidade, sobretudo no que se refere a doenças crônicas.^26^ Um estudo prévio de Giovanella et al. reforça que a facilidade de agendamento e a agilidade no atendimento da ESF são consideradas indicadores de garantia de acesso à atenção especializada.^28^

Além disso, o Protocolo Clínico e Diretrizes Terapêuticas para a DC recomendam que indivíduos com DC sejam acompanhados anualmente pela ESF para a detecção precoce da progressão clínica da doença, o tratamento e a promoção de hábitos de vida saudáveis.^29^ Quando ocorrem manifestações cardíacas graves da DC, os indivíduos devem ser encaminhados para a atenção especializada a fim de avaliar a necessidade ou o benefício do uso de dispositivos como o MP.^29^ Assim, sugere-se que, em municípios com cobertura de 100% da ESF, seja facilitada a identificação de pessoas candidatas ao uso de MP, bem como o acesso à atenção e ao acompanhamento especializados e, consequentemente, à implantação de MP.

Entre os achados, o estudo mostrou que indivíduos residentes em municípios com menor número de eletrocardiógrafos por mil habitantes disponíveis no Sistema Único de Saúde (SUS) apresentaram maior probabilidade de acesso à implantação de MP. Entre os municípios participantes do estudo, observou-se que a maioria tinha 100% de cobertura da ESF, mas também apresentava menor número de eletrocardiógrafos por mil habitantes. Isso reflete a alta cobertura da ESF, porém o baixo acesso ao exame complementar necessário. Considerando que indivíduos com DC crônica devem ser submetidos a acompanhamento longitudinal, a realização de exames complementares, como o ECG, torna-se essencial para a avaliação e o manejo iniciais.^7 , 29^ Nossa hipótese para os achados do presente estudo é que o menor número de eletrocardiógrafos dificulta o acesso a esse exame e, consequentemente, o indivíduo com DC tem a progressão do acometimento cardíaco diagnosticada tardiamente, necessitando, portanto, de intervenções, como a implantação de MP.

Quanto ao desfecho de óbito nesses grupos (caso versus controle), não houve diferença significativa nas taxas de mortalidade entre aqueles que receberam ou não a implantação de MP durante o estudo. A ausência de benefício nas taxas de sobrevida naqueles que receberam MP pode estar relacionada a diversos fatores, incluindo o tamanho da amostra, fatores de confusão não mensurados e a gravidade da cardiomiopatia. A análise de sobrevida mostrou que apenas a FEVE influenciou a sobrevida. De fato, a redução da FEVE é amplamente reconhecida como um fator independente associado ao óbito.^30^ A ocorrência de cardiomiopatia induzida por estimulação cardíaca,^31 , 32^ relacionada aos efeitos deletérios da estimulação ventricular direita crônica, também poderia estar relacionada a desfechos desfavoráveis no grupo com MP. No entanto, o presente estudo não foi delineado para avaliar essa hipótese.

### Pontos fortes e limitações

Entre os pontos fortes deste estudo, destaca-se a avaliação longitudinal de uma ampla amostra de pacientes com DC residentes em áreas endêmicas e municípios pequenos, distantes dos grandes centros urbanos comumente retratados em estudos. Isso permite a extrapolação dos resultados para outras localidades semelhantes, visto que a população com DC tipicamente apresenta um perfil epidemiológico similar.

O estudo apresenta limitações, como a homogeneidade da população em termos de características sociodemográficas. Além disso, não foi possível realizar análise multinível devido ao tamanho da amostra por município, uma vez que o estudo incluiu 184 participantes em 21 municípios. Dessa forma, as características contextuais foram analisadas como atributos individuais. Contudo, o estudo conseguiu analisar a sobrevida de indivíduos com e sem implantação de MP.

## Conclusão

Os resultados do presente estudo destacam que residir em municípios com maior população, maior cobertura da ESF e menor número de eletrocardiógrafos por mil habitantes aumentou as chances de acesso à implantação de MP. Entretanto, o MP não influenciou a mortalidade ao longo de 4 anos, a qual foi associada apenas à FEVE.

## Material suplementar

Supplementary Material 2
