# Supplementary material for: Implantação de Marca-passo em Pacientes com Doença de Chagas: Estudo Caso-Controle de Fatores Contextuais Associados e Prognóstico
Source: Arq Bras Cardiol. 2026 Mar 4;123(2):e20250003. [Article in Portuguese] doi: 10.36660/abc.20250003 (PMC13128191; doi:10.36660/abc.20250003)
Supplement: Supplementary Material 2 [file 0066-782x-abc-123-2-e20250003-suppl01.pdf]

Supplementary Material 2. Survival curves considering the following variables: left ventricular ejection fraction (LVEF), duration of the QRS complex, self-rated health perception, and physical activity practice.

A Ejection Fraction

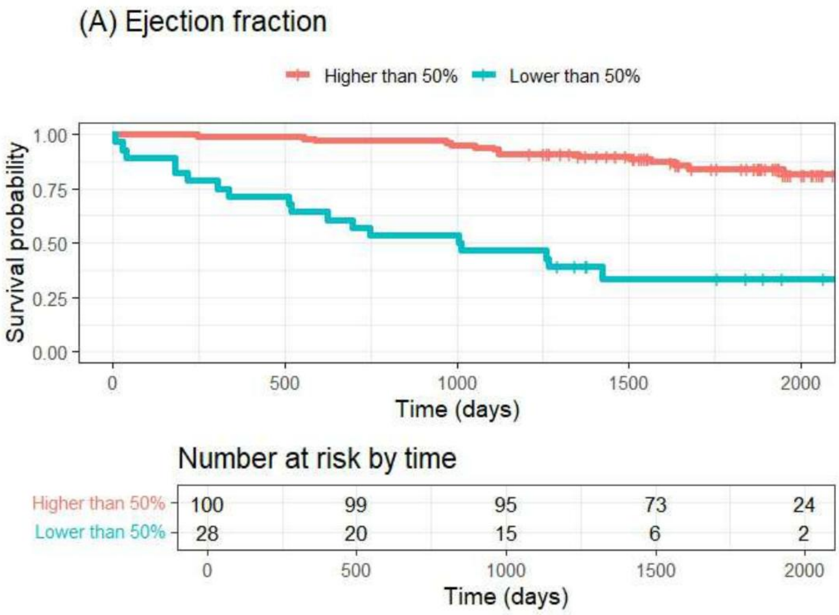

B QRS complex duration

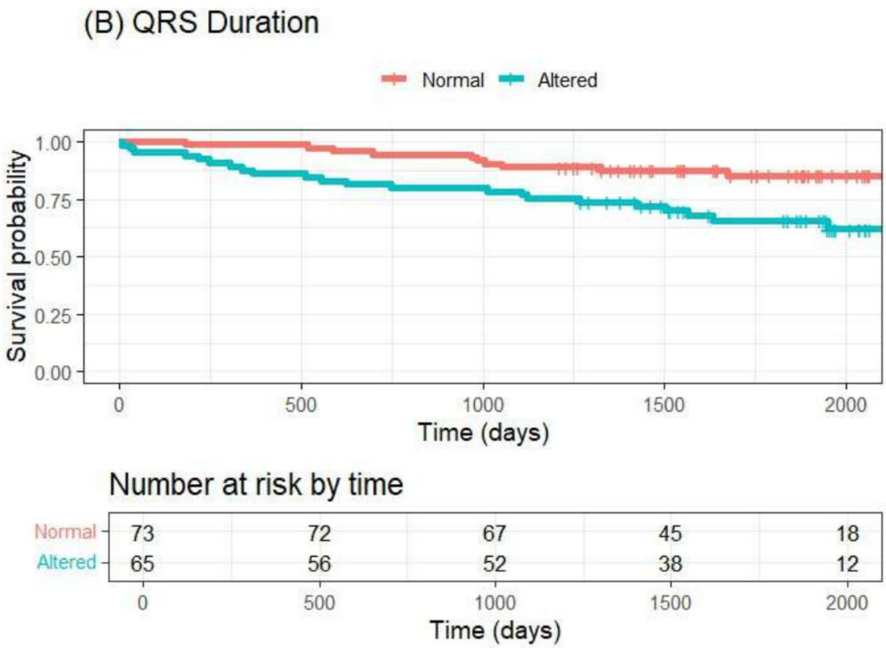

C Health perception

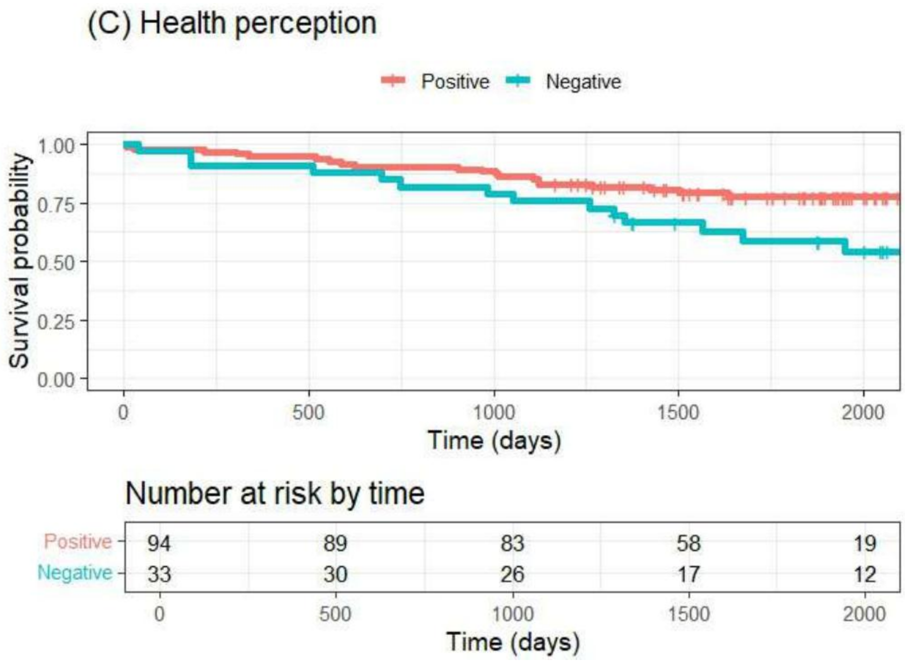

D Physical activity practice

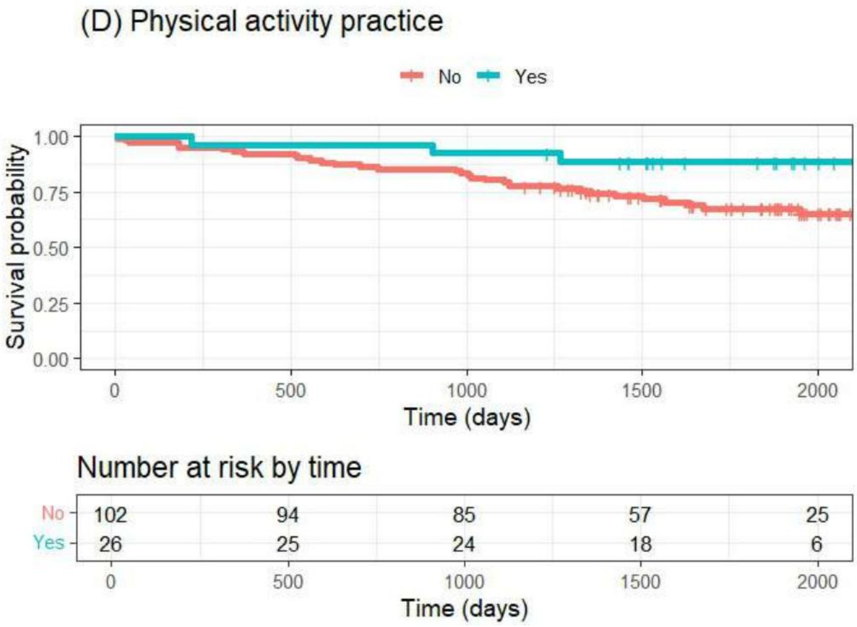

Supplementary Material 2: Survival Curve (Kaplan-Meier): **A**) Ejection Fraction (n=128*); **B**) QRS Complex Duration (n=138*); **C**) Health Perception (n=127*); and **D**) Physical Activity Practice (n=128*).
*variação no n= 159 devido perdas de informações em cada variável.
