# Supplementary material for: Implantação de Marca-passo em Pacientes com Doença de Chagas: Estudo Caso-Controle de Fatores Contextuais Associados e Prognóstico
Source: Arq Bras Cardiol. 2026 Mar 4;123(2):e20250003. [Article in Portuguese] doi: 10.36660/abc.20250003 (PMC13128191; doi:10.36660/abc.20250003)
Supplement: Translation

# Translation: Implantation of Pacemakers in Patients with Chagas disease: A Case-Control Study of Associated Contextual Factors and Prognosis

**DOI:** 10.36660/abc.20250003i

**Authors:** Sâmara Fernandes Leite, Maria Carmo Pereira Nunes, Cesar Augusto Taconeli, Mathaus Henrique Reis Silva, Dardiane Santos Cruz, Lea Campos Oliveira, Ester Cerdeira Sabino, Marco Paulo Tomáz Barbosa, Antonio Luis Pinho Ribeiro, Ariela Mota Ferreira, Desirée Sant’Ana Haikal

**Affiliations:** 1 Brazil Programa de Pós Graduação em Ciências da Saúde, Universidade Estadual de Montes Claros (UNIMONTES), MG – Brazil; 2 Brazil Departamento de Clínica Médica, Faculdade de Medicina, e Centro de Telessaúde e Serviço de Cardiologia e Cirurgia Cardiovascular, Hospital das Clínicas, Universidade Federal de Minas Gerais, Belo Horizonte, MG – Brazil; 3 Brazil Departamento de Estatística, Universidade Federal do Paraná, Curitiba, PR – Brazil; 4 Brazil Instituto de Medicina Tropical da Faculdade de Medicina da Universidade de São Paulo – USP, São Paulo, SP – Brazil; 5 Brazil Serviço de Cardiologia e Cirurgia Cardiovascular, Hospital das Clínicas, Universidade Federal de Minas Gerais, Belo Horizonte , MG – Brazil

**Keywords:** Chagas Disease, Artificial Pacemaker, Chagas Cardiomyopathy, Mortality, Survival

## Abstract

**Background:**

Chronic Chagas cardiomyopathy (CCC) is the most severe manifestation of Chagas disease and may present with bradyarrhythmias requiring pacemaker (PM) implantation.

**Objective:**

1. To identify contextual factors associated with PM implantation among individuals with CCC. 2. To assess the prognosis (death) in these groups over a 4-year follow-up period.

**Method:**

1. Nested case-control study within the SaMi-Trop cohort. We matched cases (patients with CCC who had a PM implanted between waves 1 and 2 of the study) with controls at a 1:3 ratio, considering equivalence in baseline cardiac severity. Independent variables related to the municipal context were considered. Binary logistic regression was conducted. 2. Survival analysis to evaluate the prognosis of these groups, adjusted for clinical variables (Cox regression), considering death as the outcome. Conclusions were based on a significance level of 5%.

**Results:**

The study included 46 cases and 138 controls. The odds of PM implantation were higher among individuals residing in municipalities with: larger population (odds ratio [OR]: 3.6; 95% confidence interval [CI]: 1.3 to 9.6), higher coverage of the Family Health Strategy (OR: 5.3; 95% CI: 1.8 to 15.8), and with a lower number of electrocardiographs per thousand inhabitants (OR: 2.6; 95% CI: 1.0 to 6.6). Considering 4 years of follow-up, death was observed in 32.6% of cases and 21.2% of controls. Only ejection fraction affected survival (relative risk: 7.2; 95% CI: 3.6 to 14.2).

**Conclusion:**

Contextual factors interfered with PM implantation. However, PM implantation did not influence death over 4 years of follow-up, which was associated only with ejection fraction.

## Introduction

Chagas disease (CD) is a systemic parasitic infection caused by *Trypanosoma cruzi* ( *T. cruzi* ), predominantly affecting poor and vulnerable populations.^1^It is endemic in 21 countries in Latin America, where approximately 6 million people are affected by the disease.^2^It has an annual incidence of 30,000 new cases and an annual mortality of 14,000 deaths in Latin America.^3 , 4^In Brazil, estimates range from 1.0% to 2.4% of the population infected with *T. cruzi* .^5^

Chronic Chagas cardiomyopathy (CCC) is considered the most important and severe manifestation of chronic CD.^6^It is responsible for the high morbidity and mortality of the disease, lifelong disability, as well as significant economic and social impact.^1 , 7^

CCC affects approximately 20% to 30% of individuals infected with *T. cruzi* .^8 , 9 , 10^It can present significant electrocardiographic alterations, such as second and third-degree atrioventricular block, bradycardia, atrial fibrillation,^11 , 12^as well as biochemical changes, such as the heart failure biomarker, N-terminal pro-B-type natriuretic peptide (NT-proBNP), associated with the diagnosis of CCC and different cardiomyopathies.^12 - 14^In these situations, especially for the treatment of symptomatic bradyarrhythmias and in association with other clinical evaluations, pacemaker (PM) implantation may be the therapy of choice.^6 , 7^

It is believed that contextual characteristics of the environment in which individuals with CD live can influence access to PM implantation. Furthermore, there is a scarcity in the literature of longitudinal studies that follow the progression of severely affected patients with CCC, comparing those who did and did not have access to PM implantation.

Therefore, the present study aimed to: 1. Identify contextual factors associated with PM implantation among patients with CCC, matched by the severity of cardiac involvement over 2 years of follow-up (case-control study); 2. Evaluate the relationship between PM implantation and survival in these same groups, with and without PM implantation, over 4 years of follow-up (prospective study).

## Method

### Study design

For this study, data from the SaMi-Trop cohort were used. A case-control study was conducted, followed by a prospective survival analysis.

SaMi-Trop is a multicenter cohort study involving individuals with CD, conducted through a partnership between four Brazilian public universities. It is funded by the National Institutes of Health (NIH), USA (grant numbers P50510 AI098461-02 and U19AI098461-06). The study has been following approximately 2,000 individuals with CD from 21 municipalities in two endemic regions for the disease in the state of Minas Gerais since 2013.^15 , 16^

The SaMi-Trop study comprised three waves of data collection. The first wave (baseline), which took place in 2013 to 2014, included 2,157 individuals with CD aged 18 years or older. The second wave occurred in 2015 to 2016, with 1,709 participants remaining. Finally, the third wave took place between 2019 and 2021, with 1,115 participants remaining. In all assessments, interviews, blood collection, and 12-lead electrocardiogram (ECG) were performed, and echocardiogram examination was added in the follow-ups. The interview covered sociodemographic data, lifestyle habits, physical activity, aspects of quality of life, medical history, and CD treatment.

### Ethical aspects

Ethical approval was obtained from the appropriate ethics committee (CEP/USP 042/2012, UNIMONTES 2.474.172, and CONEP 179.685). All individuals agreed to participate in this study and signed the informed consent form before the study began.

### Case-control study (first objective)

#### Selection of participants

Using data from the SaMi-Trop cohort participants, two analysis groups were formed (case versus control), considering only as potential participants those individuals with confirmed serology for CD via enzyme-linked immunosorbent assay (ELISA) who presented cardiac involvement in the first wave of evaluation (criteria presented below).

“Case” individuals were defined as those who underwent PM implantation between the first and second waves of evaluation. “Control” individuals were defined as those who did not undergo PM implantation between the first and second waves of evaluation but had cardiac involvement in the first wave, similar to the cases (matching).

A 1:3 (case:control) manual matching of individuals was performed considering the variables of cardiac involvement, sex (male versus female), and age categorized by the mean (up to 60 years versus 61 years or older). Cardiac involvement was defined as: second-degree atrioventricular block and third-degree atrioventricular block, atrial fibrillation, sinus bradycardia, all categorized as no versus yes; functional class categorized as no alteration (class 1) versus with alteration (classes 2, 3, and 4) according to the New York Heart Association (NYHA),^17^and NT-proBNP level categorized by age (unaltered versus altered).^13^

#### Dependent variable

The dependent variable was related to the implantation of PM between the first and second follow-up waves (case versus control). It was obtained from interview questions and confirmed through ECG.

#### Contextual and municipal data

Data collection from the context was conducted to characterize the social, demographic, and health services aspects of the 21 municipalities participating in the SaMi-Trop cohort. Eight contextual variables were considered, collected from public health information platforms and systems. The information was collected with reference to the year of the cohort’s first wave of evaluation (2013 to 2014), seeking the closest available data to this reference. Supplementary Material 1 presents information about the contextual variables considered.

The variables Municipal Human Development Index (MHDI) and Unified Health System Performance Index (IDSUS) were collected and categorized according to international and national standards and subsequently dichotomized. The cutoff points adopted were “low” (0 to 0.55) versus “medium/high” (0.556 to 0.699/0.700 to 0.799) and “0.50 to 0.59” versus “0.60 to 0.79” for the MHDI and IDSUS, respectively. The variables population, Gini index, total health expenditure per capita, Family Health Strategy (FHS) coverage, distance from the reference center, and number of electrocardiographs per thousand inhabitants were collected numerically and later dichotomized, adopting the 25th percentile or the 75th percentile as the cutoff point, depending on whether the variable represented a negative measure (low values indicating better situation) or positive measure (high values indicating better situation). The aim was to separate 25% of the municipalities in better situations from 75% of the municipalities in worse situations, given that the included municipalities generally presented similar and mostly precarious profiles ( Table 1 ). The variable presence of cardiologist was collected and categorized as present versus not present.

**Table 1:** – Distribution of matching variables between case (with pacemaker) and control (without pacemaker) groups. Patients with Chagas disease. Minas Gerais, Brazil (2013 to 2016)

Characteristics	Descriptive	Bivariate		p value**
	n (%)	Control n=138	Case n=46	
**Matching variables** Second-degree AV block*				
Negative	180 (98.4%)	136 (98.6%)	44 (97.8%)	0.723
Positive	3 (1.6%)	2 (1.4%)	1 (2.2%)	
Third-degree AV block*				
Negative	182 (99.5%)	138 (100%)	44 (97.8%)	0.079
Positive	1 (0.5%)	0 (0.0%)	1 (2.2%)	
AF*				
Negative	176 (96.2%)	132 (95.7%)	44 (97.8%)	0.519
Positive	7 (3.8%)	6 (4.3%)	1 (2.2%)	
Sinus bradycardia*				
Negative	176 (96.2%)	133 (96.4%)	43 (95.6%)	0.803
Positive	7 (3.8%)	5 (3.6%)	2 (4.4%)	
**Functional class**				
No functional class alteration	78 (42.4%)	58 (42%)	20 (43.5%)	0.863
Functional class with alteration (2, 3, and 4)	106 (57.6%)	80 (58%)	26 (56.5%)	
NT-proBNP level*				
Not altered	140 (76.5%)	104 (75.9%)	36 (78.3%)	0.745
Altered	43 (23.5%)	33 (24.1%)	10 (21.7%)	
**Sex**				
Male	64 (34.8%)	47 (34.1%)	17 (37%)	0.721
Female	120 (65.2%)	91 (65.9%)	29 (63%)	
**Age**				
Up to 60 years	87 (47.3%)	67 (48.6%)	20 (43.5%)	0.551
61 years and older	97 (52.7%)	71 (51.4%)	26 (56.5%)	

AF: atrial fibrillation; AV: atrioventricular. * Total n variation of 184. ** Pearson’s chi-square test.

#### Statistical analysis

Descriptive and bivariate analysis (Pearson’s chi-squared test) of the matching variables was conducted to confirm the similarity between case and control groups. The effect of PM implantation on participants’ survival time was analyzed using survival analysis techniques. The Kaplan-Meier method was used to adjust survival curves and conduct preliminary comparison of groups, and the log-rank test to compare the survival times.

Subsequently, multiple modeling was initiated using traditional binary logistic regression. All contextual variables were initially included in the analysis, and income, literacy, and self-reported skin color variables were kept as adjustment factors. Contextual variables were adjusted using the backward stepwise method manually, considering a significance level of 5%. The Hosmer-Lemeshow test was used to assess the goodness of fit of the logistic model. The percentage of variance explained by the model was estimated using Nagelkerke’s Pseudo R^2^. The analyses were conducted using Predictive Analytics SoftWare (PASW/SPSS)^®^, version 24.0 for Windows^®^.

## Prospective study (second objective)

### Study subjects

The same analysis groups selected in the second wave of evaluation were considered: case (with PM implantation) versus control (without PM implantation). Individuals lost to follow-up and individuals from the control group who underwent pacemaker implantation after the second wave of evaluation were excluded from the analyses.

### Dependent variable

The event of interest was death (no versus yes). The dependent variable considered was the time elapsed from the second wave of data collection until death occurred or until the end of the follow-up period (third wave), approximately 2,200 days (6 years).

Death was considered for individuals for whom this information was made available (family members or health services) or a record was identified in the National Mortality System, where searches were carried out for all lost participants.

### Independent variables

For the independent variables, the case versus control categorization established in this study was considered, along with a set of clinical, sociodemographic, and behavioral variables, including left ventricular ejection fraction (LVEF) obtained from echocardiogram and categorized as: normal (≥ 50%) versus altered (< 50%).^18^The variable of QRS complex duration was categorized as: normal (n < 120 ms) versus altered (≥ 120 ms).^18^Diabetes mellitus and hypertension remained as collected: no versus yes. Self-perceived health was obtained from the participant’s self-report during the interview through the question “How would you rate your health today?” A Likert scale was adopted with response options and subsequently dichotomized as: positive (good, very good, and fair) versus negative (poor and very poor). Per capita income was categorized into half the minimum wage in effect at the time of data collection (2014) as: above BRL 362.01 versus below BRL 362.00. Literacy was assessed with the question, “Can you read and write?” categorized as: no versus yes. Marital status was categorized as: stable union (cohabiting with partner, married) versus no stable union (single, widowed, and others). Physical activity was assessed with the question “Do you practice any physical activity?” and categorized as: no versus yes.

## Statistical analysis

The effect of PM implantation on participants’ survival time was analyzed using survival analysis techniques. The Kaplan-Meier method was used to adjust survival curves and conduct preliminary comparison of groups. Subsequently, regression analysis was performed to estimate and test the effect of interest. In this stage, the Cox proportional hazards model was used, considering, in addition to PM implantation status, the following set of variables: ejection fraction, QRS duration, diabetes, hypertension, self-perceived health, income, literacy, marital status, and physical activity. Additionally, the effect of the experimental design (case-control) was incorporated into the model through a random effect (frailty term) with a gamma distribution, in order to accommodate within-group correlation and between-group variability induced by the design. Initially, bivariate analysis was conducted to assess the effect of PM implantation and other variables not adjusted by the others on survival time. Variables significant at the 20% level were considered for inclusion in the multiple regression model. After adjusting the multiple regression model, non-significant variables at the 5% level were successively removed from the model. Results are presented in the form of relative risks (RR) and 95% confidence intervals (CI), along with their respective significances. The interaction between treatment (PM) and ejection fraction was also investigated to assess possible differences in treatment effect for participants with different levels of ejection fraction. Conclusions were based on a significance level of 5%. All analyses were performed using the statistical software R for statistical computing, version 4.2.1. The survival library was used for survival analysis.^19^

## Results

Among the 1,709 individuals with CD evaluated in the second wave of the SaMi-Trop cohort, 46 cases and 138 controls were identified, totaling 184 individuals ( Figure 1 ).

**Figure 1:** – Flowchart of participants classified as case and control, less to follow-up, and occurrence of death.

Considering the matching variables, there was no statistical difference between case and control ( Table 1 ), indicating similarity between the groups.

### First objective

Among the contextual variables considered for assessing the association with PM implantation, it was observed that the majority of individuals resided in municipalities with a smaller population (67.4%), with higher MHDI (78.8%), with higher Gini index (91.3%), with better IDSUS (66.3%), lower total health expenditure per inhabitant (89.7%), with greater coverage of the FHS (57.6%), without a cardiologist in the health service (81%), farther from the specialized health reference center (87.5%), and with fewer electrocardiographs per thousand inhabitants (65.8%).

The adjusted multiple regression model revealed a higher likelihood of PM implantation among those who resided in municipalities with a larger population (OR: 3.655; 95% CI: 1.385 to 9.650), in municipalities with greater FHS coverage (OR: 5.341; 95% CI: 1.805 to 15.804), and in municipalities with a lower number of electrocardiographs per thousand inhabitants (OR: 2.6; 95% CI: 1.057 to 6.620) ( Table 2 ). Contextual factors were associated with access to PM implantation ( Central Illustration ).

**Table 2:** – Multiple binary logistic regression model of contextual factors associated with the implantation of artificial pacemakers in individuals from the SaMi-Trop cohort. Minas Gerais, Brazil. n = 184

			Contextual factors associated with pacemaker implantation	
Variables	Control	Case	OR [95% CI]	p value
**Population**				
Smaller population	96 (69.6%)	28 (60.9%)	1	0.009
Larger population	42 (30.4%)	18 (39.1%)	3.655 [1.385-9.650]	
**FHS coverage**				
Less than 100% coverage	62 (44.9%)	16 (34.8%)	1	0.002
100% coverage	76 (55.1%)	30 (65.2%)	5.341 [1.805-15.804]	
**Number of electrocardiographs per thousand inhabitants**				
Above 0.21	49 (35.5%)	14 (30.4%)	1	0.038
Below 0.20	89 (64.5%)	32 (69.6%)	2.645 [1.057-6.620]	
Hosmer-Lemeshow test p value = 0.513 Pseudo R^2^(Nagelkerke): 10.3% Model adjusted for income, literacy, and skin color				

FHS: Family Health Strategy.

### Second objective

In the second objective, the death of 15 (32.6%) individuals among the cases and 24 (21.2%) among the controls was observed ( Figure 1 ). Among the individuals classified as controls, there were 25 losses, and thus the participation of 113 controls was considered in this analysis (second objective).

Figure 2 displays the survival curves for the case and control groups (p > 0.05).

**Figure 2:** – Kaplan-Meier Survival Curve of Case vs Control Groups.

In the bivariate analysis regarding death, variables such as LVEF, QRS duration, and physical activity were associated with the outcome. In the Cox regression, only the LVEF variable (RR: 7.164; 95% CI: 3.615 to 14.197) significantly affected the survival of individuals with CD ( Central Illustration ). The PM was not associated with the outcome ( Table 3 ).

**Table 3:** – Bivariate analysis and multiple Cox regression model of the relationship between pacemaker implantation and prognosis (death) in the groups, over 4 years of follow-up. Endemic regions of the State of Minas Gerais, Brazil. n = 113

Covariates	Relative risk [95% CI]	p value	Multiple regression model, relative risk [95% CI]	p value
**Categorization control and case**				
Case	1.437 [0.717 - 2.877]	0.425	1.408 [0.710-2.793]	0.333
**Ejection fraction**				
Altered	7.164 [3.615- 14.197]	≤ 0.001	7.201[3.632-14.277]	≤ 0.001
**QRS duration**				
Altered	2.350 [1.103 -5.007]	≤ 0.001	-	-
**Diabetes**				
Yes	0.853 [0.310-2.347]	0.997	-	-
**Hypertension**				
Yes	1.126 [0.518-2.449]	0.927	-	-
**Health perception**				
Negative	1.903 [0.926-3.908]	0.061	-	-
**Income**				
Below BRL 362.00	0.975 [0.475-2.002]	0.738	-	-
**Literate**				
Yes	0.709 [0.339-1.481]	0.222	-	-
**Marital status**				
Stable union	1.185 [0.569-2.467]	0.755	-	-
**Physical activity practice**				
	0.293 [0.086-0.996]	0.027	-	-

Supplementary Material 2 presents the survival curves considering the following variables: LVEF, duration of the QRS complex, self-rated health perception, and physical activity practice.

The log-rank test was used to compare the survival times between groups. According to the test results, no difference was observed between the survival times of patients in the case and control groups (p = 0.403). However, significant differences were identified for different levels of ejection fraction (p < 0.001), health perception (p = 0.010), QRS duration (p = 0.048), and physical activity (p = 0.045). The survival curves estimated by the Kaplan-Meier method, showed in Supplementary Material 2, indicated longer survival times for patients with LVEF above 50%, positive health perception, normal QRS duration, and those who engaged in physical activities.

The interaction between PM and LVEF was also investigated to assess a possible difference in treatment effect for patients with different levels of ejection fraction. However, no significant effect was observed for this interaction (p = 0.39).

## Discussion

The results of this study demonstrated that, when matching individuals with CD and similar cardiac involvement, contextual factors were associated with access to PM implantation. Residing in municipalities with larger populations, municipalities with greater coverage of the FHS, and municipalities with fewer electrocardiographs per thousand inhabitants increased the likelihood of accessing PM implantation. Furthermore, when following these same individuals with CD and similar cardiac involvement, it was observed that having or not having access to PM implantation did not influence mortality over 4 years. Mortality was only associated with altered LVEF.

Despite the recognized relevance of contextual factors in chronic diseases,^19 , 20 , 21^there is a gap in the literature regarding PM implantation in patients with chronic CD, especially considering the characteristics of the context in which they live. The influence of variables reflecting health conditions on PM implantation has already been established in the literature.^10^However, it is necessary to consider that information related to the context in which one lives influences patients’ health conditions.^19 - 21^

It was observed that residing in municipalities with a larger population increased the chance of access to PM implantation among participants. This finding was initially expected because larger municipalities tend to have easier access to specialized health care and tend to have better health and care indicators.^23 , 24^

Individuals residing in municipalities with higher FHS coverage had a greater chance of accessing PM implantation. The FHS plays a crucial role in managing patients with CD, as it is strategically positioned in relation to other points of health care.^25^Because it is the closest health service to this population and is present in rural and remote areas, it can be considered a key player in patient care, facilitating access to specialized care and follow-up for these patients, especially in less favored areas.^26 , 27^The FHS absorbs a large part of the health demands of a community, especially regarding chronic diseases.^26^A previous study by Giovanella et al. reinforces that the ease of scheduling and the agility in FHS care are considered indicators of guaranteed access to specialized care.^28^

Moreover, the Clinical Protocol and Therapeutic Guidelines for CD recommend that individuals with CD be monitored annually by the FHS for early detection of the disease’s clinical progression, treatment, and promotion of healthy lifestyle habits.^29^When severe cardiac manifestations of CD occur, individuals should be referred to specialized care to assess the need for or benefit of using devices such as PM.^29^Thus, it is suggested that in municipalities where there is 100% coverage of the FHS, the identification of people who are candidates for PM is facilitated, as well as access to specialized care and follow-up, and consequently, PM implantation.

Among the findings, the study showed that individuals residing in municipalities with a lower number of electrocardiographs per thousand inhabitants available in the Brazilian Unified Health System (SUS) increased the chance of accessing pacemaker implantation. Among the participating municipalities in the study, it was observed that most had 100% coverage of the FHS but also presented a lower number of electrocardiographs per thousand inhabitants. This reflects that there is high FHS coverage but low access to the necessary complementary examination. Considering that individuals with chronic CD should undergo longitudinal follow-up, the performance of complementary exams, such as ECG, becomes essential for initial evaluation and management.^7 , 29^Our hypothesis for the finding of this study is that the lower number of electrocardiographs hinders access to this exam, and consequently, the individual with CD has the progression of cardiac involvement diagnosed late, thus requiring interventions, such as PM implantation.

Regarding the outcome of death in these groups (case versus control), there was no significant difference in death rates between those who received or did not receive the PM implant during the study. The absence of benefit in survival rates in those who received a PM may be related to several factors, including the sample size, unmeasured confounders, and the severity of the cardiomyopathy. The survival analysis showed that only LVEF influenced survival. Indeed, reduced LVEF is widely recognized as an independent factor associated with death.^30^The occurrence of pacing-induced cardiomyopathy,^31 , 32^related to deleterious effects of chronic right ventricular pacing, could also be related to unfavorable outcomes in the PM group. However, this study was not designed to evaluate this hypothesis.

### Strengths and limitations

Among the strengths of this study, the longitudinal assessment of a large sample of patients with CD living in endemic areas and small municipalities, far from the major urban centers commonly portrayed in studies, stands out. This allows for the extension of results to other similar locations, given that the population with CD typically has a similar epidemiological profile.

The study has limitations, for example, homogeneity of the population in terms of sociodemographic characteristics. Moreover, it was not possible to perform multilevel analysis due to the sample size per municipality, as we have 184 participants in 21 municipalities. Thus, contextual characteristics were analyzed as individual attributes. However, the study was able to analyze the survival of individuals with and without PM implantation.

## Conclusion

The results of this study highlight that living in municipalities with larger populations, higher coverage of FHS, and fewer electrocardiographs per thousand inhabitants increased the chances of accessing PM implantation. However, the pacemaker did not influence mortality over 4 years, which was only associated with LVEF.

## Supplemental Materials

Supplementary Material 2
